# Impact of Maneuverability Constraints on Intraoral Scanner Performance

**DOI:** 10.3390/diagnostics16030501

**Published:** 2026-02-06

**Authors:** Chieh-Ming Yu, Wei-Chun Lin, Chiao-Yun Peng, Chian-Chuen Lee, Chia-Cheng Lin

**Affiliations:** 1 Department of Dentistry, Shin Kong Wu Ho-Su Memorial Hospital, Taipei 111, Taiwan; jaminzeak@hotmail.com (C.-M.Y.); chiaoyun2023@gmail.com (C.-Y.P.); 2 School of Dental Technology, College of Oral Medicine, Taipei Medical University, Taipei 110, Taiwan; weichun1253@tmu.edu.tw (W.-C.L.); b210110036@tmu.edu.tw (C.-C.L.); 3 Department of Dentistry, Wan-Fang Hospital, Taipei Medical University, Taipei 116, Taiwan

**Keywords:** accuracy, digital intraoral scanning, scan time, trueness, intraoral scanner

## Abstract

**Background/Objectives:** Intraoral scanners (IOSs) are essential tools in digital dentistry; however, their accuracy remains influenced by clinical conditions such as restricted access, patient movement, or intraoral moisture. Intraoral scanning is performed within a confined space that restricts scanner motion, potentially influencing maneuverability during data acquisition and, consequently, IOS performance. This study investigated the impact of maneuverability constraints on the trueness accuracy and efficiency of IOS under clinically representative intraoral conditions. **Methods**: Fifteen participants with no previous experience in intraoral scanning or device operation were recruited. Each participant scanned a maxillary full-dentition typodont model and a mandibular implant-containing typodont model using the Aoralscan 3 IOS. Scans were performed under two conditions: constrained intraoral scanning within a manikin and open-vision extraoral scanning on a bench-top. Trueness accuracy was evaluated using three parameters: the root mean square (RMS) deviation of the maxillary dentition, discrepancies in inter–scan body distances, and angular deviations of the scan bodies, each calculated by comparison with reference data obtained from an industrial-grade scanner. Scan time was recorded to assess time-based efficiency. **Results**: No significant differences were observed in RMS trueness, inter-implant distances, or implant angular deviations between intraoral and extraoral scans. Extraoral scanning significantly reduced scan times for both maxillary and mandibular models (*p* < 0.0001). **Conclusions**: Within the limitations of this study, maneuverability constraints alone may not significantly affect IOS trueness accuracy compared with open bench-top scanning. However, scanning efficiency was reduced under intraoral scanning constraints, with longer scan times observed among inexperienced operators. The potential influence of intraoral factors other than maneuverability on IOS accuracy under clinical conditions warrants further investigation.

## 1. Introduction

Dental impression is an indispensable clinical procedure to restorative dentistry, as they provide the geometric information required for diagnosis, treatment planning, and fabrication of dental restorations [1,2]. Despite being the standard of care for an extended period, conventional impressions exhibit notable limitations, including dimensional changes in impression materials and dental stones, time-consuming, patient discomfort, and sensitivity to operator technique [3,4,5]. To address these drawbacks, optical scanners and digital design and manufacturing technologies were introduced. In particular, intraoral scanner (IOS) has emerged as a key innovation, offering a reliable and fully digital alternative to conventional impression techniques. Digital impressions captured with IOS enable the generation of accurate digital models, streamline clinical workflows, and enhance patient comfort [6,7,8].

Despite the significant advantages offered by IOS, several limitations remain that affect their clinical applicability. A primary concern is the reduced accuracy observed during full-arch or long-span scans, where cumulative stitching errors can lead to distortion of the digital model [9,10,11]. High reflectivity from metallic surfaces or saliva accumulation, along with restricted intraoral access, can impair scan quality and compromising data accuracy [1,12,13]. However, IOSs can also be applied for the digitization of stone casts, and this extraoral approach has been widely adopted in several in vitro and ex vivo investigations. Clinically acceptable accuracy has been reported and, in many cases, improved precision compared with intraoral scanning has been observed, largely due to the absence of confounding clinical factors such as saliva contamination, patient movement, and restricted access [14,15,16]. Kernen et al. [17] demonstrated reduced intraoral scanning accuracy using the same experimental appliance scanned extraorally and intraorally. They concluded that intraoral scanning accuracy was reduced under clinical conditions compared with extraoral scanning; however, the specific intraoral factors contributing to this difference were not individually investigated, and scanner maneuverability was not explicitly considered.

Intraoral scanning inherently imposes constraints on scanner movement and orientation due to the confined oral environment, which may disrupt scanning trajectories and compromise image overlap during sequential data acquisition. In contrast, scanning performed under open, direct-vision conditions—such as extraoral scanning of the same object—allows greater freedom of scanner motion and more stable acquisition paths. These differences in physical access and working space influence scanner maneuverability during data capture and may represent a critical, yet insufficiently investigated, factor affecting IOS performance.

Therefore, the purpose of this study was to investigate the impact of maneuverability constraints on the trueness accuracy and time-based efficiency of IOS performance by comparing scanning outcomes obtained in a clinically representative intraoral setting with those obtained under extraoral scanning on a bench-top. The null hypotheses of this study were as follows: There would be no significant differences in the trueness accuracy and scanning efficiency of IOS between clinically representative intraoral scanning and open-vision extraoral scanning on a bench-top.

## 2. Materials and Methods

**Figure 1:**
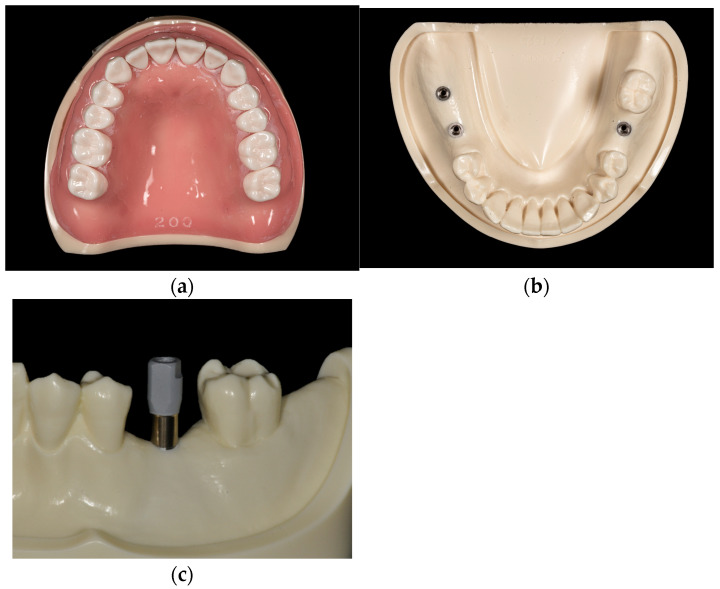
Typodont models used in this study: (**a**) maxillary model with full dentition; (**b**) mandibular model with implants positioned in the edentulous posterior sites corresponding to teeth 47, 46, and 36; (**c**) titanium scan body attached to tooth 36. Tooth designations: 36 (mandibular left first molar), 46 (mandibular right first molar), and 47 (mandibular right second molar).

To minimize variables commonly encountered in clinical settings, such as tongue interference and the presence of saliva, a controlled simulation environment was established using a dental laboratory manikin. A maxillary typodont model (U200, Nissin Dental Products Inc., Kyoto, Japan) was mounted in occlusion with a mandibular model (P9-X.1523-L, Nissin Dental Products Inc., Kyoto, Japan), in which three implants (Kyocera POIEX, 3.7 × 10 mm; Kyocera Medical Co., Osaka, Japan) with pre-attached titanium scan bodies (GEO CAD, Geomedi Co., Ltd., Fukuoka, Japan) were installed in the edentulous posterior regions corresponding to teeth 47 (right second molar), 46 (right first molar), and 36 (left first molar). The scan body incorporated an orientation marker and a surface coating optimized for spray-free optical scanning (Figure 1). The vertical distance between the central incisors of the two models was adjusted to 45 mm, reflecting the average maximum mouth opening typically observed in Asian populations [18].

A total of fifteen participants, all dental technology students with no prior experience using IOS, were recruited for this study. The IOS used for the simulation was the Aoralscan 3 (Shining 3D Tech Co., Hangzhou, China), which was calibrated prior to the experiment. Before scanning, all participants received standardized instruction on IOS operation and were asked to follow a predetermined scanning strategy starting from the posterior teeth in the sequence: buccal → occlusal →lingual. Each operator completed two sets of maxillary and mandibular scans, one under constrained intraoral conditions within a manikin and the other under extraoral bench-top conditions with direct visual access. The total time taken for each scan was also recorded as a measure of scan efficiency. Because participants inevitably gained experience during the first scanning procedure, scanning sequences were counterbalanced, with eight participants starting intraorally and seven starting extraorally, to distribute this improvement evenly across conditions.

**Figure 2:**
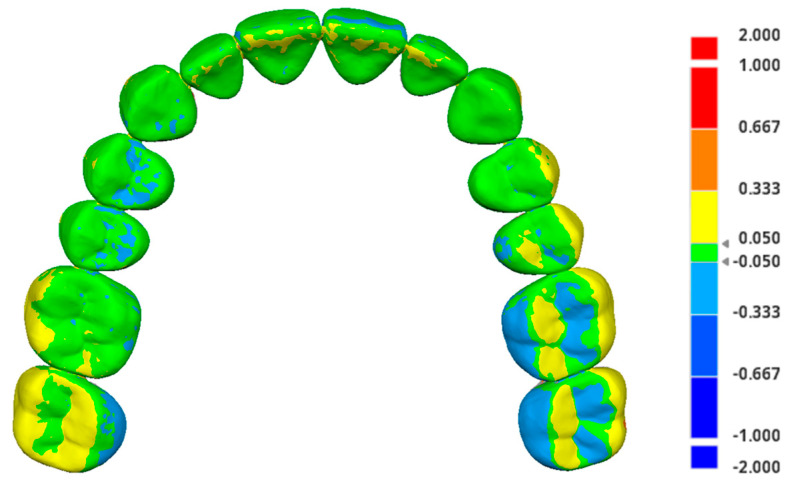
Color-coded deviation map illustrating the differences between the IOS-acquired scan and the reference model. The color scale indicates the magnitude and direction of deviations in millimeters.

The IOS-acquired images of the maxillary and mandibular models were saved as Standard Tessellation Language (STL) files and compared with a reference digital master model generated using a high-precision industrial scanner (ATOS 5; Zeiss GOM GmbH, Braunschweig, Germany). For the maxillary model, each IOS-generated STL file, along with the master model, was imported into Medit Design software (Version 2.1.4.97, Medit Corp., Seoul, South Korea). Using the Smart Single Tooth Selection feature, soft tissue regions were excluded from each maxillary scan. The datasets were then automatically aligned to the master model using the software’s automatic alignment option. Root mean square (RMS) trueness values were determined by assessing the deviations between the IOS data and the reference model within the Deviation Display Mode (Figure 2).

**Figure 3:**
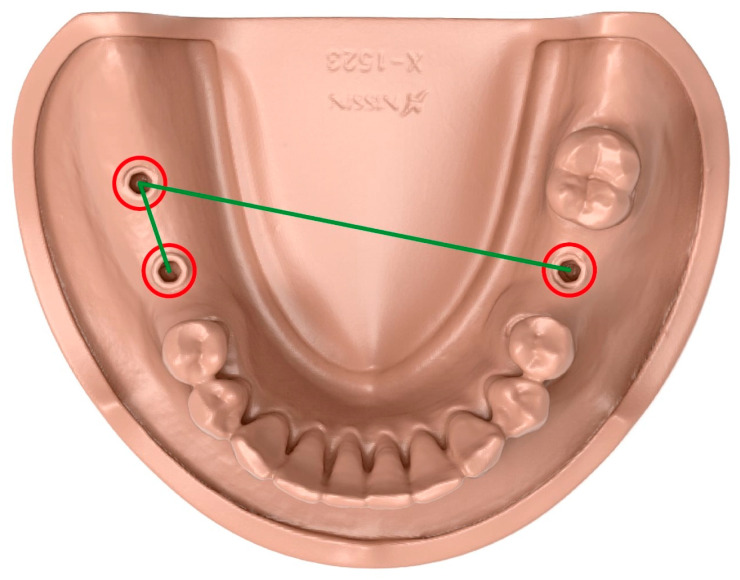
Inter-scan-body distance measurements were used to assess intraoral scanner accuracy. The three-dimensional distances between the centers of the upper openings of the scan bodies at positions 46–47 and 36–47 were calculated, and deviations from the reference values were used as indicators of trueness accuracy.

For the mandibular model, each IOS-generated STL file, along with the master model, was imported into GOM Inspect software (2022 Service Pack 2, Rev.157456, Carl Zeiss GOM Metrology GmbH, Braunschweig, Germany). The three implant scan bodies were identified using the actual elements function. The datasets were first automatically aligned to the master model using the software’s initial alignment function, followed by best-fit alignment. Using the inspection tool, two parameters were evaluated. First, the three-dimensional inter-scan-body distances between the centers of the upper openings of the scan bodies at positions 46–47 and 36–47 were determined, and discrepancies relative to the master model were calculated. Second, the angular deviations between the long axes of the corresponding scan bodies in the master model and the IOS scans were quantified (Figure 3).

In this study, the trueness accuracy was evaluated based on three parameters: the RMS value of the maxillary dentition, the discrepancies in inter-scan body distances, and the angular deviations of the scan bodies. Based on preliminary data from a pilot study, a sample size of 15 scans per group was determined to be sufficient to detect a moderate effect size (0.5) with 80% statistical power and a significance level (α) of 0.05. All statistical analyses were conducted using SAS software (version 9.4; SAS Institute, Cary, NC, USA). Quantitative outcomes are reported as mean values with corresponding standard deviations. Between-group comparisons were performed using independent-samples *t*-tests or Mann–Whitney U tests, as appropriate, depending on the distributional characteristics of the data. A *p*-value of less than 0.05 was considered statistically significant.

## 3. Results

**Figure 4:**
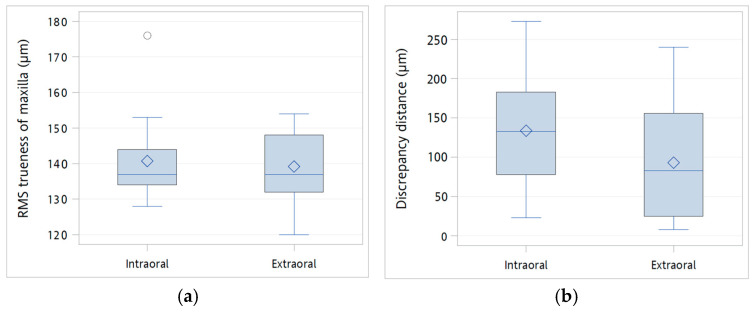

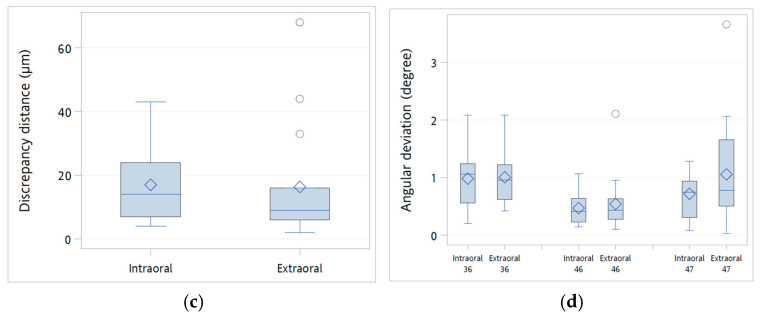
Box plots comparing IOS trueness accuracy under intraoral (*n* = 15) and extraoral (*n* = 15) scanning conditions: (**a**) Root mean square (RMS) trueness of the maxillary dentition; (**b**) discrepancy in the inter–scan-body distance between the centers of the upper openings at positions 36–47; (**c**) discrepancy in the inter–scan-body distance between positions 46–47; and (**d**) angular deviation of the scan bodies at positions 36, 46 and 47. The horizontal line within each box represents the median, the diamond indicates the mean, and circles denote outliers. The box corresponds to the interquartile range (IQR), and the whiskers indicate the overall range of the data.

The accuracy of the IOS used both intra- and extraorally was presented in Table 1 and Figure 4. The RMS trueness measured for the maxillary dentition demonstrated no significant difference between the two scanning scenarios. Specifically, the intraoral scanning yielded an RMS value of 141 ± 12 μm, while the extraoral scanning achieved a closely comparable value of 139 ± 10 μm, with a *p*-value of 0.7015, indicating equivalence in performance for this anatomical region.

For the mandible, a smaller discrepancy was observed in the 36–47 inter-implant distance during extraoral scanning (93 ± 72 μm) compared to intraoral scanning (134 ± 72 μm), however, this reduction did not reach statistical significance (*p* = 0.1343, independent *t*-test; *p* = 0.1197, Mann–Whitney U test). The discrepancy in the 46–47 inter-implant distance was comparable between intraoral scans (17 ± 12 μm) and extraoral scans (16 ± 18 μm), with no significant difference detected (*p* = 0.908, independent *t*-test; *p* = 0.4061, Mann–Whitney U test). Similarly, no significant differences were observed in angular deviations at teeth 36 (*p* = 0.9091, independent *t*-test; *p* = 0.9504, Mann–Whitney U test), 46 (*p* = 0.6278, independent *t*-test; *p* = 0.9835, Mann–Whitney U test), or 47 (*p* = 0.2046, independent *t*-test; *p* = 0.2259, Mann–Whitney U test).

**Figure 5:**
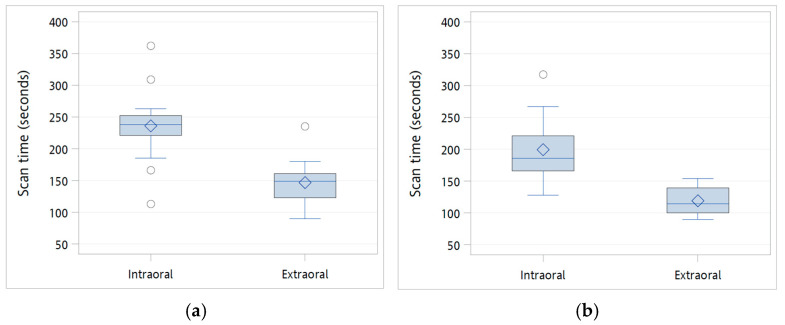
Box plots of scanning times performed intraorally (*n* = 15) and extraorally (*n* = 15): (**a**) maxilla and (**b**) mandible. The horizontal line within each box represents the median, the diamond indicates the mean, and circles denote outliers. The box corresponds to the interquartile range (IQR), and the whiskers indicate the overall range of the data.

In contrast, a notable difference was observed in the time-based efficiency of the scans, as summarized in Table 2 and Figure 5. The extraoral scanning method significantly reduced scan times for both the maxilla and mandible compared to intraoral scanning. For the maxilla, intraoral scanning required 236.0 ± 56.9 s, compared to 146.7 ± 36.9 s for extraoral scanning (*p* < 0.0001, independent *t*-test; *p* = 0.0001, Mann–Whitney U test). Similarly, mandibular scanning times were reduced for extraoral scans compared with intraoral scans (199.3 ± 50.1 s vs. 119.1 ± 20.2 s; *p* < 0.0001, independent *t*-test and Mann–Whitney U test).

## 4. Discussion

Given the multitude of factors inherent to the intraoral environment that can influence IOS performance, it is intuitive to anticipate superior scanning outcomes when the same device is used under extraoral conditions. Kernen et al. [17] employed a custom-designed reference appliance incorporating artificial teeth and fiducial elements to compare intraoral and extraoral scanning. The appliance was digitized intraorally after placement in the oral cavity and subsequently scanned outside the mouth using IOSs, enabling direct comparison between scanning environments. Their results demonstrated lower accuracy and precision for intraoral scans than for extraoral scans, leading the authors to conclude that clinical scanning conditions adversely affected IOS performance. They noted that factors such as confined intraoral space, moisture, and the characteristics of IOS image acquisition could have influenced data capture; however, these factors were discussed collectively, and no individual variable was isolated or examined in detail.

Previous literature has primarily discussed intraoral scanner maneuverability as a design- or ergonomics-related consideration, often in relation to scanner tip size, access to posterior regions, and operator handling, rather than as a direct determinant of scanning accuracy [19,20]. Therefore, this study aimed to investigate whether maneuverability constraints influence the trueness accuracy and scanning efficiency of IOSs. Direct comparison between intraoral and extraoral IOS scans is inherently challenging, because dentition present in the oral cavity can only be reproduced extraorally through indirect procedures, such as conventional impression taking and stone cast fabrication followed by digitization. This multistep workflow introduces additional sources of error, complicating direct intraoral–extraoral accuracy comparisons [21]. To address this limitation, the present study evaluated maneuverability constraints using a manikin setup designed to simulate clinically representative intraoral conditions.

The results of this study showed that, with respect to trueness accuracy, no statistically significant differences were identified between constrained intraoral scanning and open-vision extraoral scanning across all evaluated parameters, indicating that the null hypothesis regarding accuracy could not be rejected. In contrast, scanning efficiency was significantly reduced under intraoral conditions, with longer scan times observed; therefore, the null hypothesis regarding scanning efficiency was rejected. Taken together, these findings indicate that maneuverability constraints primarily affect the efficiency of IOS use rather than trueness accuracy under the controlled conditions examined in this study. Within this context, restricted maneuverability alone did not result in reduced IOS trueness accuracy when other intraoral variables were controlled. This observation suggests that intraoral factors beyond maneuverability—such as surface reflectivity, optical interference from moisture, lighting conditions, and soft-tissue movement, which were not isolated in the present study—may play a more prominent role in IOS accuracy degradation under clinical conditions and warrant further investigation. The efficiency of extraoral scanning can be attributed to the unobstructed visualization and easier accessibility of the scanned object, which enable more ergonomic and controlled manipulation of the scanner. This advantage appears to be particularly beneficial for inexperienced operators, who may otherwise encounter greater difficulty during intraoral scanning.

Operator experience has been reported as a factor that may influence IOS scanning accuracy, and several studies have indicated improved performance with increasing operator experience [22,23,24,25,26]. Therefore, this study intentionally recruited novice operators to perform the test scans. The inclusion of experienced operators could have resulted in improved intraoral scanning performance, potentially compensating for maneuverability constraints and thereby reducing the sensitivity to detect the specific influence of maneuverability on IOS performance. Notably, because no difference in trueness accuracy was observed between intraoral and extraoral scanning even among inexperienced operators, it is reasonable to infer that maneuverability constraints alone are unlikely to exert a greater impact on trueness accuracy when scans are performed by more experienced operators. However, this inference may not be directly applicable to the efficiency outcomes of the present study, as experienced operators are generally expected to perform scans more efficiently than inexperienced operators. Consequently, the magnitude of the observed differences in scanning efficiency between intraoral and extraoral conditions may be attenuated when scans are performed by experienced users. Nevertheless, it should be noted that intraoral scanning in clinical settings often requires additional time due to patient-related factors within the oral cavity, such as limited mouth opening, challenges in moisture control, existing restorations, and soft-tissue movement [27]. As a result, the actual difference in scanning efficiency may be more pronounced when IOS is used in real clinical scenarios.

In their investigation, Gimenez et al. [28] reported that IOS accuracy may differ among operators, although this variation does not necessarily reflect differences in experience. When only a few operators are involved, individual skill levels may disproportionately affect outcomes. To minimize this influence, we enrolled 15 participants to perform the simulation scans. This strategy minimized the influence of individual operator skill, thereby enhancing the generalizability of the findings. However, the inclusion of operators with varying handling characteristics increased within-group variability, as reflected by the relatively large standard deviations observed in inter–scan-body distance discrepancies and angular deviations. Although the sample size of 15 was necessarily limited by the experimental design, it was determined a priori based on pilot data to ensure adequate sensitivity for detecting meaningful differences. In addition, both parametric and non-parametric statistical analyses were applied according to the distributional characteristics of the data, thereby reducing potential bias associated with distributional assumptions. The consistency of results obtained across these analytical approaches supports the internal validity of the statistical findings despite the inherent constraints of sample size.

Zarauz et al. [23] reported that age may affect scanning performance among inexperienced operators, with older users potentially requiring longer training to achieve improved accuracy, while advances in IOS software may help enhance the accuracy obtained by novice users. In the present study, the inexperienced operators were senior dental technology students who, although they had not previously operated an IOS clinically, had received theoretical instruction regarding IOS principles and scanning workflows and possessed a foundational understanding of dental anatomy. In addition, as young individuals familiar with information technology, they were able to adapt quickly to IOS operation. Therefore, despite their limited hands-on experience, the scanning procedure was not particularly difficult for this participant group, which may explain the satisfactory accuracy achieved in this study.

IOSs generate three-dimensional (3D) models through the sequential integration of multiple overlapping images acquired from different perspectives. The scanning strategy is therefore closely associated with the performance of image-stitching algorithms and may influence the overall accuracy of IOSs [14,29]. Latham et al. [30] reported that scan pattern may affect the trueness and precision of complete-arch digital scans, although the impact varies among different intraoral scanners. Adherence to the manufacturer’s recommended scanning strategy may yield the highest accuracy, with significantly better complete-arch impression trueness compared with alternative scanning patterns [29]. In the present study, the scanning sequence was initiated from the buccal surface of the posterior region rather than the occlusal surface, because the occlusal aspect of posterior implant scan bodies lacks distinctive morphological features for reliable image registration. Because all scans followed the same predefined scanning strategy, its influence on the outcomes of this study was minimized. Nevertheless, the effect of scanning strategy on scan body acquisition accuracy warrants further investigation.

Previous studies have demonstrated that IOSs can achieve accuracy comparable to laboratory scanners in limited scenarios, such as single-tooth or single-quadrant scans; however, performance tends to decline in full-arch or cross-arch applications, largely due to cumulative stitching errors during image integration [31,32,33]. For full-arch or high-precision reference applications, dedicated desktop laboratory scanners remain the preferred option, as they generally provide equal or superior accuracy and efficiency and are therefore frequently used as reference devices in digital dentistry research [34,35,36]. In the present study, multiple accuracy parameters were selected to capture potential differences across both shorter and longer scanning spans. In addition to RMS trueness evaluation of the maxillary dentition, both adjacent and cross-arch implant scan body–based measurements were included. Although no statistically significant differences were observed across the evaluated trueness parameters, the possibility remains that subtle effects were not fully detected, particularly for the 36–47 inter-implant distance. In the mandible, a smaller discrepancy was observed for this parameter during extraoral scanning compared with intraoral scanning (93 ± 72 μm vs. 134 ± 72 μm). Given that implant scan body acquisition introduces additional geometric and procedural complexity, maneuverability constraints may become more relevant in clinically challenging scanning tasks, such as implant-supported restorations or prepared abutment scans. Further studies are necessary to clarify whether maneuverability plays a greater role under these more demanding conditions.

Only one IOS system was evaluated in the present study. However, a wide range of IOS devices are currently available, incorporating different distance-acquisition technologies, including confocal microscopy, active triangulation, and active wavefront sampling [37]. In addition, modern IOS systems increasingly integrate artificial intelligence algorithms capable of automatically detecting and removing soft-tissue artifacts. AI-based functions such as smart stitching, which facilitate data integration during image reconstruction, have also been reported to enhance scanning efficiency [6]. Therefore, the findings of this study may not be directly generalizable to all IOS platforms. Moreover, the scanner hardware characteristics—such as tip dimensions and accessibility—may indirectly influence scanning performance, especially in posterior segments. In most studies, maneuverability is discussed primarily in terms of ease of use, clinical feasibility, and patient comfort rather than as an isolated determinant of quantitative accuracy outcomes. For example, Fratila et al. [19] noted that scanner tip size and geometry may restrict maneuverability in posterior regions, particularly in patients with limited mouth opening or anatomical constraints. In the present study, however, regional deviations between anterior and posterior areas were not separately evaluated, and therefore potential location-dependent effects could not be identified. Future studies using additional IOS systems and region-specific accuracy analyses are needed to better elucidate the role of maneuverability and scanner design across different intraoral regions.

Several additional limitations should be acknowledged. Trueness assessment was evaluated based on RMS deviation as this study was designed as an exploratory evaluation of the influence of maneuverability constraints on IOS performance. Detailed parameters underlying RMS computation, such as alignment settings, iteration limits, and convergence thresholds, were not systematically explored. Moreover, more comprehensive accuracy analyses—such as localized deviation assessment, cumulative stitching error visualization, or region-specific error breakdowns (e.g., anterior versus posterior)—were not performed, limiting the depth of the investigation. Precision was not evaluated in the present study because the inclusion of multiple operators introduces substantial interpersonal variability, making repeated-scan precision comparisons across participants less informative. Moreover, as an in vitro investigation, the present study has inherent limitations in terms of clinical applicability. Future studies incorporating experienced operators and repeated scan acquisitions are warranted to better characterize operator-related variability, repeatability, and cumulative stitching error accumulation in full-arch IOS workflows. Despite these limitations, the findings of this study indicate that IOS trueness accuracy can be maintained under clinically representative maneuverability constraints, whereas restrictions on scanner movement have a measurable impact on scanning efficiency. These results highlight maneuverability as an important process-related factor influencing IOS performance and suggest that it should be considered when interpreting intraoral scanning outcomes in clinical contexts.

## 5. Conclusions

Within the limitations of this study, the following conclusions can be drawn:

1. Maneuverability constraints alone may not result in measurable accuracy differences when compared with open bench-top scanning, as assessed by RMS trueness of the dentition, inter-implant distances, and angular deviations.
2. In contrast, scanning efficiency was significantly affected by maneuverability constraints, with open bench-top scanning demonstrating shorter scanning times than intraoral scanning, particularly among inexperienced operators.
3. Potential influence of other intraoral factors beyond maneuverability, such as surface reflectivity, moisture, and soft-tissue movement, may contribute more substantially to accuracy degradation in clinical environments and warrant further investigation.

## Figures and Tables

**Table 1:** Trueness accuracy of intraoral and extraoral scanning.

	Intraoral	Extraoral	*p*-Value (Independent *t*-Test)	*p*-Value (Mann–Whitney U Test)
RMS of maxillary dentition (μm)	141 ± 12	139 ± 10	0.7015	0.9503
Discrepancy in 36–47 distance (μm)	134 ± 72	93 ± 72	0.1343	0.1197
Discrepancy in 46–47 distance (μm)	17 ± 12	16 ± 18	0.908	0.4061
Angular deviation of 36 (degree)	0.98 ± 0.57	1.01 ± 0.52	0.9091	0.9504
Angular deviation of 46 (degree)	0.47 ± 0.29	0.54 ± 0.49	0.6278	0.9835
Angular deviation of 47 (degree)	0.72 ± 0.36	1.06 ± 0.93	0.2046	0.2259

36: mandibular left first molar, 46: mandibular right first molar, 47: mandibular right second molar.

**Table 2:** Scan time (seconds) of intraoral and extraoral scanning.

	Intraoral	Extraoral	*p*-Value (Independent *t*-Test)	*p*-Value (Mann–Whitney U Test)
Maxilla	236.0 ± 56.9	146.7 ± 36.9	<0.0001 *	0.0001 *
Mandible	199.3 ± 50.1	119.1 ± 20.2	<0.0001 *	<0.0001 *

* Significant at *p* < 0.05.

## Data Availability

The data that support the findings of this study are not publicly available due to ethical and privacy considerations.
